# Genome‐wide methylation profiling of diagnostic tumor specimens identified DNA methylation markers associated with metastasis among men with untreated localized prostate cancer

**DOI:** 10.1002/cam4.6507

**Published:** 2023-09-11

**Authors:** Chun R. Chao, Jeff Slezak, Kimberly Siegmund, Kimberly Cannavale, Yu‐Hsiang Shu, Gary W. Chien, Xu‐Feng Chen, Feng Shi, Nan Song, Stephen K. Van Den Eeden, Jiaoti Huang

**Affiliations:** ^1^ Department of Research and Evaluation Kaiser Permanente Southern California Pasadena California USA; ^2^ Department of Health Systems Science Kaiser Permanente Bernard J Tyson School of Medicine Pasadena California USA; ^3^ Department of Population and Public Health Sciences, USC Keck School of Medicine University of Southern California Los Angeles California USA; ^4^ Department of Urology, Los Angeles Medical Center Kaiser Permanente Southern California Los Angeles California USA; ^5^ Department of Pathology, School of Medicine Duke University Durham North Carolina USA; ^6^ Department of Pathology, Beijing Shijitan Hospital Capital Medical University Beijing China; ^7^ Department of Urology Beijing Shijitan Hospital Capital Medical University Beijing China; ^8^ Division of Research Kaiser Permanente Northern California Oakland California USA

**Keywords:** epigenetics, metastasis, methylation, prostate cancer, risk model

## Abstract

**Background:**

We used a genome‐wide discovery approach to identify methylation markers associated with metastasis in men with localized prostate cancer (PCa), as better identification of those at high risk of metastasis can inform treatment decision‐making.

**Methods:**

We identified men with localized PCa at Kaiser Permanente California (January 1, 1997–December 31, 2006) who did not receive curative treatment and followed them for 10 years to determine metastasis status. Cases were chart review‐confirmed metastasis, and controls were matched using density sampling. We extracted DNA from the cancerous areas in the archived diagnostic tissue blocks. We used Illumina's Infinium MethylationEPIC BeadChip for methylation interrogation. We used conditional logistic regression and Bonferroni's correction to identify methylation markers associated with metastasis. In a separate validation cohort (2007), we evaluated the added predictive utility of the methylation score beyond clinical risk score.

**Results:**

Among 215 cases and 404 controls, 31 CpG sites were significantly associated with metastasis status. Adding the methylation score to the clinical risk score did not meaningfully improve the c‐statistic (0.80–0.81) in the validation cohort, though the score itself was statistically significant (*p* < 0.01). In the validation cohort, both clinical risk score alone and methylation marker score alone are well calibrated for predicted 10‐year metastasis risks. Adding the methylation score to the clinical risk score only marginally improved predictive risk calibration.

**Conclusion:**

Our findings do not support the use of these markers to improve clinical risk prediction. The methylation markers identified may inform novel hypothesis in the roles of these genetic regions in metastasis development.

## Graphical abstract

We performed genome‐wide methylation interrogation of diagnostic prostate tissue of localized prostate cancer. We identified methylation markers at 31 CpG sites that significantly predicted risk of metastasis using Bonferroni correction. However, incorporating these 31 methylation markers in risk prediction models did not significantly improve risk prediction for metastasis beyond clinical risk factors.

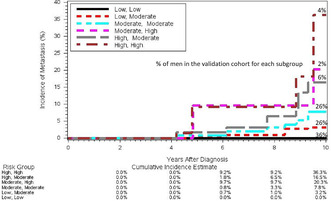

## 1 INTRODUCTION

In 2022, 268,490 men are estimated to be diagnosed with prostate cancer (PCa) in the United States.^1^ Of these, about 80% are localized.^2^ A significant proportion of localized PCa is indolent and will not affect a patient's health even in the absence of curative treatment.^3^ On the other hand, PCa treatment often leads to persistent side effects that compromise patient's quality of life.^4^ The American Urology Association (AUA) treatment guidelines recommended active surveillance for very low‐risk PCa, and as a management option for low‐risk and favorable intermediate‐risk PCa.^5^ Distinguishing the subset of localized PCa cases whose metastasis may be prevented by treatments is critical to inform treatment decision‐making.

Established algorithms for predicting indolent PCa are based on clinical and pathological features at diagnosis.^6^, ^7^, ^8^, ^9^, ^10^, ^11^, ^12^, ^13^ These prediction algorithms have been found to misclassify a proportion of patients in validation studies.^6^, ^14^, ^15^ Routine clinical and pathological features are unlikely to fully capture the complex molecular mechanisms underlying tumor behavior. These existing prediction tools also offer little insight for potential molecular mechanisms for aggressive PCa. Epigenetic regulation is now recognized as a major mechanism underlying tumor heterogeneity and disease progression.^16^, ^17^ DNA methylation is a stable and readily measurable epigenetic modification.^18^ The contribution of aberrant DNA methylation to metastatic process has become evident.^16^, ^17^, ^19^ We thus hypothesize that certain methylation markers will be predictive of metastasis in men with localized PCa and can enhance the current risk prediction algorithm for PCa aggressiveness.

A major challenge of studying molecular tumor markers for PCa aggressiveness is the need to focus on specimens obtained at the time of diagnosis rather than at surgery because surgery may alter the disease course (e.g., by preventing or delaying metastasis in aggressive PCa). It is thus impossible to classify the pretreatment risk of metastasis in treated patients. In a recent systematic review,^20^, ^21^ most studies that examined the associations between DNA methylation markers and PCa outcome focused on treated patients. The majority of the studies also employed a candidate gene approach,^22^ which may hinder the development of novel risk prediction scores based on the most informative methylation markers. To shed light on the utility of DNA methylation markers on risk prediction for PCa aggressiveness, we conducted a nested case–control study to identify novel methylation markers measurable at PCa diagnosis that is predictive of PCa metastasis among untreated men using a discovery approach and examined the added predictive value of these methylation markers beyond clinical risk factors.

## 2 METHODS

### 2.1 Study setting, design, and populations

This study was conducted at Kaiser Permanente Southern California (KPSC) and Northern California (KPNC), integrated health care delivery systems that provide comprehensive health services for over 9‐million racial and socioeconomically diverse members who are broadly representative of residents in California.^23^, ^24^ We first identified the source cohort based on the following criteria: (1) newly diagnosed with PCa between January 1, 1997, and December 31, 2006 at KPSC or KPNC; and (2) diagnosed at localized stage (TNM stages I and II or localized stage of the SEER summary stage). We then excluded men who: (1) had unknown Gleason score; (2) received prostatectomy, radiation therapy, hormone therapy, or chemotherapy within 6 months of diagnosis; and (3) had metastatic disease, died, or were lost to follow‐up within 6 months of diagnosis. We followed all eligible men from 6 months after PCa diagnosis until metastasis, initiation of prostatectomy, radiotherapy, chemotherapy or immunotherapy (since treatment will alter the disease course), death (non‐PCa‐related), health plan disenrollment, or 10 years after initial PCa diagnosis (~88% of the men who remained alive at 10 years after PCa diagnosis retained KP membership), whichever came first.

We included all men with identified metastasis during study follow‐up as cases. We selected controls from the source cohort using density sampling, individually matched to cases at a 3:1 ratio initially by age (within 5 years), race (black vs. nonblack), year of PCa diagnosis (within 5 years), and KP region (i.e., KPSC or KPNC). We later sampled additional controls to allow proper adjustment for Gleason score in the analysis.

We also included a separate validation cohort for the evaluation of the added predictive utility of DNA methylation markers discovered from the case–control set. We included men diagnosed with localized PCa in 2007 at KPSC who met the other eligibility criteria described above and followed them as above. Because metastasis risk prediction was most helpful for those with Gleason score 7 or lower (i.e., treatment is recommended for those with Gleason score 8 or higher), we further limit the validation cohort to those with Gleason score ≤7.

Both the KPSC's and KPNC's Institutional Review Boards approved this study and waived the requirement for informed consent. We conducted this study in accordance with the U.S. Common Rule guidelines.

### 2.2 Data collection

We identified men diagnosed with PCa from KPSC's and KPNC's Surveillance, Epidemiology, and End Result‐affiliated cancer registries. The KP cancer registries collect data on clinical stage, Gleason score (Gleason grade was not collected for all study years, therefore it was not used), age at diagnosis, race/ethnicity as well as initial course of cancer treatment. We identified potential metastases using an algorithm based on ICD‐9 and ICD‐10 diagnosis codes for metastatic malignancy, natural language processing of radiology reports, serum PSA levels >20 ng/mL, CPT procedure codes for treatment initiation and utilizations of chemotherapy, immunotherapy, or hormonal therapy (leuprolide), encounters with an oncologist after 12 months following diagnosis, and death due to prostate cancer. We manually chart reviewed probable cases identified by the algorithm to confirm prostate cancer metastasis and date of metastasis diagnosis. We re‐reviewed a random sample of 10% of the charts to ensure the quality of the chart reviewed. An experienced urologist (GWC) reviewed all questionable cases and made a final determination on the presence of metastasis.

We identified PCa treatment and PSA levels from KP's electronic health records and date and cause of death from KP's records and death certificate linkage with the State of California and Social Security Administration.

### 2.3 Pathology review

We retrieved archived diagnostic biopsy H&E slides and formalin‐fixed, paraffin‐embedded (FFPE) blocks for study subjects from KP's centralized storage center. Previous studies have shown strong correlations comparing methylation values between paired FFPE and fresh‐frozen tumor tissues using Illumina's genome‐wide methylation technology^25^, ^26^ and supported the use of FFPE sample for our purpose. The study pathologists (JH, FS, and NS) centrally reviewed all pathology reports and diagnostic slides to identify appropriate biopsy cores for the methylation assay and circled the cancerous areas on each FFPE block for macrodissection. We choose not to perform microdissection for the following reasons: (1) Gene expression studies suggest an important role of stromal cells in the metastasis signature^27^, ^28^; (2) microdissection is limiting for routine clinical use due to costs; and (3) PCa tumor glands are usually clustered in biopsy cores, making macrodissection easy to perform and contamination less of a concern. When necessary, we used multiple biopsy cores to achieve sufficient DNA sample for a subject for the methylation assays.

### 2.4 Laboratory procedures

We performed DNA extraction using Qiagen's QIAamp DNA FFPE Tissue Kit. Methylation assay was performed at the University of Southern California Core Facility using Illumina's Infinium MethylationEPIC BeadChip, following manufacturer protocol. The technician first bisulfite converted the purified DNA (Zymo's EZ DNA methylation kit) and treated them with a FFPE DNA Restoration Kit before the assay. Methylation status of the interrogated CpG site is presented as the *β* value, which is a continuous variable ranging from 0 (unmethylated) to 1 (fully methylated). We included each case–control trio in the same batch and same BeadChip and randomly assigned well positions within a case–control trio. The USC core lab performed background correction and dye‐bias normalization using the “noob” function in the minfi R package.^29^

### 2.5 Statistical analysis

We calculated the distributions of the demographic and clinical characteristics of the final analytical case–control set and the validation cohort and compared the cases and controls using the Wilcoxon rank‐sum test or chi‐square test. We estimated the 10‐year cumulative incidence of metastasis in the source cohort and the validation cohort using the Kaplan–Meier method.

For the analysis of the methylation data, we omitted the data points with probe failure rate >5% (33,697 probes, or 3.9% of all probes), leaving 832,140 probes in the analyses. We did not further filter methylation signals that may be due to SNPs or genomic deletions or copy number variations given our purpose was mainly prediction. Using the methylation data from the case–control set, we performed conditional logistic regressions to test the association between methylation status (i.e., the *β* value) of each of the 832,140 CpG sites (after excluding those with >5% probe failure) in the MethylationEPIC BeadChip and metastasis status (i.e., the case status) after adjusting for age at diagnosis, race, Gleason score, and LUMP score. A separate model was constructed for each CpG site. We adjusted for the LUMP score (leukocytes unmethylation for purity, which averaged 44 non‐methylated immune‐specific CpG sites to infer tumor purity), to account for potential tissue impurities in the DNA samples.^30^ We defined genome‐wide significance as *p* value less than 1 × 10^−7^ based the considerations of (1) Bonferroni correction of *α*‐level 0.1 (0.1/865,837 = 1.17 × 10^−7^, with *α* = 0.10 to avoid over restriction in the search of predictors); and (2) balance between established *p* value cutoffs for GWAS studies (5 × 10^−8^ and 5 × 10^−7^)^31^ We generated the Manhattan plot of minus log *p* values by position in base pairs for each chromosome to evaluate the relative positions of the significant CpG sites after Bonferroni correction (Figure S1).

We initially created two sets of methylation scores based on the CpG sites identified: The first score was the weighted sum of *β* values for each selected site, weighted by the regression coefficient estimates from the individual logistic regression; the second score used only the sign of the regression coefficient (positive or negative) and is the “sum” of the *β* values for the sites (with the *β* values for CpG sites with negatives associations subtracted instead of added). We compared the area under the ROC curve (AUC) for the risk prediction model including each of the methylation scores in the case–control dataset. The two scores achieved the same AUC, thus the score with coefficient signs as weights was chosen due to its simplicity. We also created a single clinical risk score (composed of age at diagnosis, race/ethnicity, stage at diagnosis, Gleason score, PSA at diagnosis, PSA doubling time, and number of positive biopsy cores) rather than adjusting for multiple clinical variables in the model, which allowed us to condense clinical risk factors into a single degree of freedom for modeling. This clinical risk score was created in the case/control set using logistic regression. Some of the clinical predictors (i.e., PSA doubling time and number of biopsy cores positive) had considerable missingness (up to 20%). We therefore used multiple imputation with the chained equation technique for all the analyses.^32^

We evaluated the AUC with the clinical risk score alone, the methylation score alone, and both the clinical risk score and the methylation score in both the case–control set (using conditional logistic regression) and in the validation cohort (using Cox regression) to assess the predicted risk discrimination of each of these models. In subset analyses, we repeated the analyses for those with Gleason score of 7 vs. ≤6. In addition, we assessed predicted risk calibration in the validation cohort by comparing model‐predicted risk groups (<5%, 5%–14.9%, and 15%+) of the 10‐year risk of metastasis and observed risk (estimated by 10‐year cumulative incidence) for both the model with clinical risk score alone and the model with both the clinical and methylation risk scores. There was not enough sample size to perform stratified analysis on predicted risk calibration by Gleason score. We performed the methylation data processing and EWAS analysis using R, version 4.3.1. We conducted all other analyses using the SAS Enterprise Guide statistical software, version 7.1.

## 3 RESULTS

### 3.1 Study population

**FIGURE 1:**
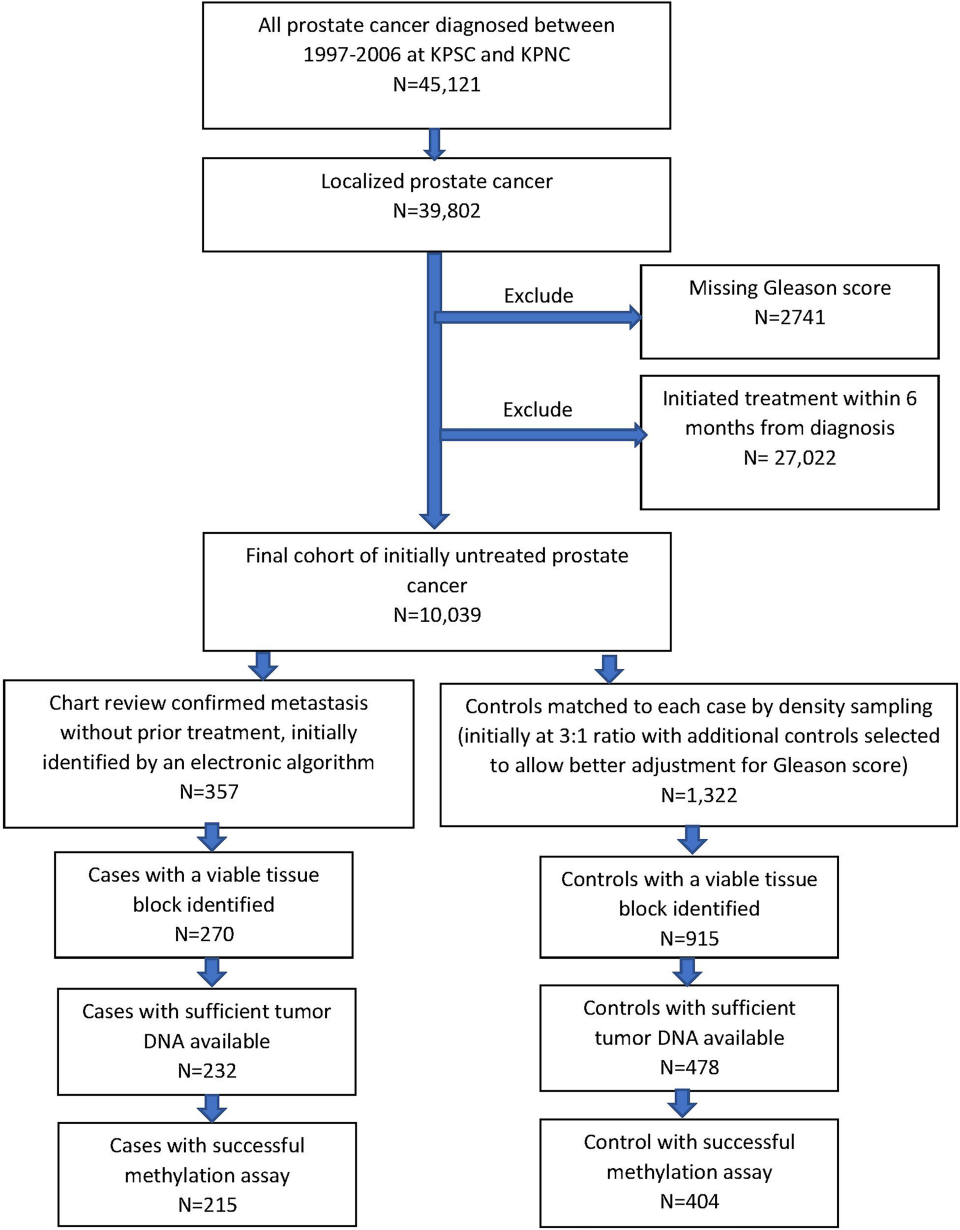
Study population flow chart–case–control set.

A total of 39,802 men diagnosed with localized PCa between 1997 and 2006 were identified at KPSC and KPNC. After applying the exclusion criteria, the source cohort for the case–control selection consisted of 10,039 men (Figure 1). Of these men, a total of 357 metastases that developed within 10 years after diagnosis were chart review confirmed, with a total of 1322 controls selected. Among the identified cases and controls, a total of 215 cases and 404 controls contributed successful methylation results (Figure 1).

For these 215 cases and 404 controls, the mean age at PCa diagnosis was 72.7 and 72.9 years, respectively. About 42% and 26% of the cases had Gleason score 7 and 2–6, respectively, while 27% and 51% of the controls had Gleason score 7 and ≤6, respectively. Cases also had higher level of PSA at diagnosis, shorter PSA doubling time, and number of biopsy cores positive for cancer (Table 1).

**TABLE 1:** Demographic and clinical characteristics of the study populations.

	Cases (*N* = 215)	Controls (*N* = 404)	*p* Value*	Validation cohort with methylation data (*N* = 382)
Age (mean [SD])	72.7 (8.8)	72.9 (8.4)	0.74	67.0 (9.9)
Race/ethnicity				
White	122 (56.7%)	248 (61.4%)	0.15	190 (49.7%)
Hispanic	27 (12.6%)	31 (7.7%)		70 (18.3%)
Black	46 (21.4%)	77 (19.1%)		78 (20.4%)
Asian	14 (6.5%)	26 (6.4%)		26 (6.8%)
Others	6 (2.8%)	22 (5.4%)		18 (4.7%)
Year of diagnosis				
1997–1999	56 (26.1%)	117 (29.0%)	0.84	
2000–2002	60 (27.9%)	111 (27.5%)		
2003–2006	99 (46.0%)	176 (43.6%)		
2007				382 (100%)
Stage at diagnosis				
I	208 (96.7%)	399 (98.8%)	0.12	378 (98.9%)
II	7 (3.2%)	5 (1.2%)		4 (1.1%)
Gleason score				
≤6	56 (26.0%)	207 (51.2%)	<0.01	262 (68.6%)
7	90 (41.9%)	109 (26.8%)		120 (31.4%)
≥8	69 (32.1%)	91 (22.5%)		0 (0%)
PSA at diagnosis, (mean [SD])	34.9 (86.2)	14.8 (25.1)	<0.01	7.6 (5.65)
PSA doubling time				
Negative or stable	81 (37.7%)	191 (46.9%)	0.01	104 (27.2%)
<3 years	51 (23.7%)	71 (17.4%)		96 (25.1%)
≥3 years	27 (12.6%)	71 (17.4%)		117 (30.6%)
Missing	56 (26%)	74 (18.2%)		65 (17.0%)
Number of biopsy positive cores, (mean [SD])	5.9 (3.38)	4.7 (3.02)	<0.01	3.2 (2.47)

* *p* Value from Wilcoxon rank‐sum test for continuous variables and chi‐square test for categorical variables.

**FIGURE 2:**
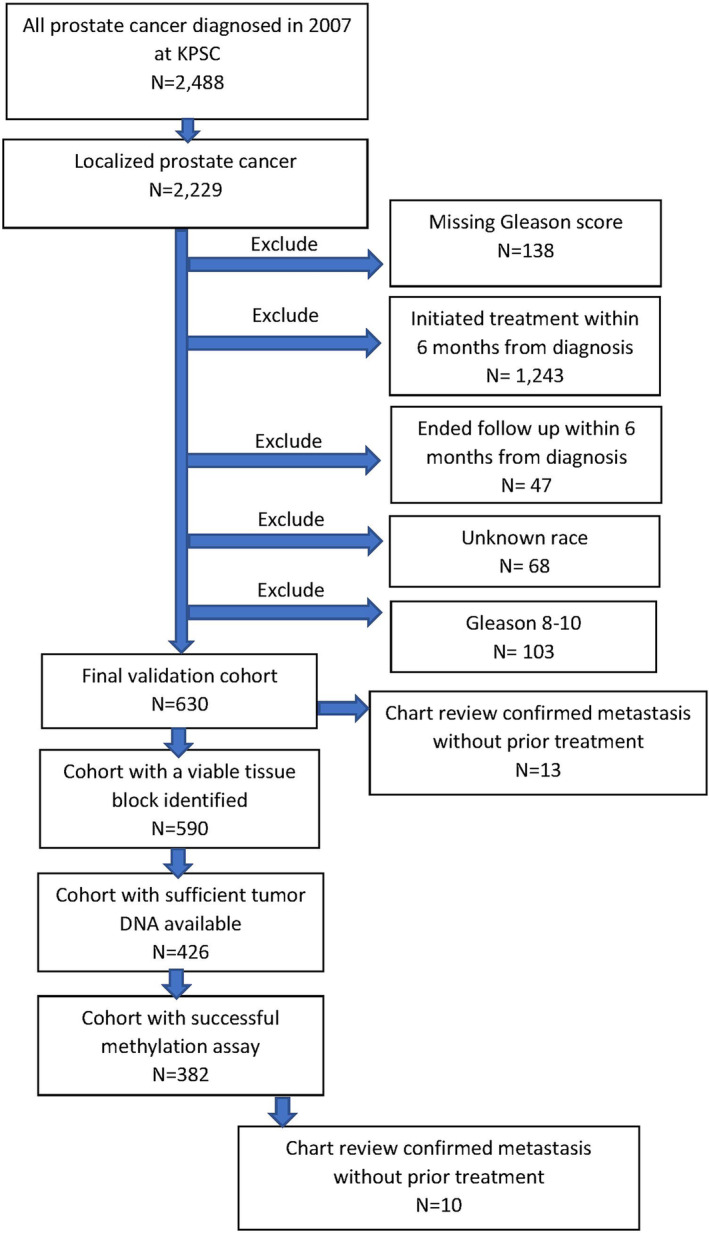
Study population flow chart–validation set.

A total of 2229 men diagnosed with localized PCa in 2007 were identified at KPSC; 630 met all eligibility criteria for the validation cohort, and 382 contributed successful methylation results (Figure 2, including 10 cases who developed metastasis (3 with Gleason score ≤6 and 7 with Gleason score 7)). The mean age at diagnosis in the final validation cohort was 67.0 years. Thirty‐one percent and 69% of the validation cohort had Gleason score 7 and ≤6, respectively (Table 1, right).

### 3.2 Cumulative incidence of metastasis

In the source cohort, the cumulative incidence of metastasis was 7.8% over 10 years. The 10‐year cumulative incidence varies by the index calendar year, ranging from 3.8% (1997) to 10.8% (1998) without a clear trend over time. In the validation cohort, the 10‐year cumulative incidence was 8.0%.

### 3.3 Selection of methylation markers

Analysis of the MethylationEPIC BeadChip data among the cases and controls led to the identification of 31 CpG sites whose methylation status was significantly associated with metastasis status after Bonferroni correction. These 31 sites, their associations with PCa metastasis (measured as odds ratio) and *p* values are listed in Table 2.

**TABLE 2:** The 31 CpG sites identified by Bonferroni adjustment and their odds ratio for metastasis and *p* values.

CpG site (Illumina ID)	Chromosome	Gene	Relation_to_UCSC_CpG_Island	OR	*p* Value
cg03347306	1	PRDM16;PRDM16	Body	0.009404	1.42E‐08
cg09104747	1	RP11‐423O2.7		0.00509	8.79E‐08
cg17113117	1	chr1:2772126‐2772665 (CpG island name)	N_Shelf	0.007875	3.23E‐08
cg21472468	1	chr1:146522288‐146522555	Shore	0.00346	1.73E‐08
cg23883265	2	AC097532.1	Low‐CpG	0.002525	1.18E‐08
cg04278212	3	chr3:129855195‐129855560		0.002029	5.07E‐09
cg08679466	3	ALDH1L1‐AS2	Body	0.027	8.00E‐08
cg16030580	3	RP11‐379F4.4;MFSD1		55.19584	5.33E‐08
cg24718437	3	AC090954.1		0.001628	6.26E‐08
cg05085627	4	AC116562.1		0.008198	1.21E‐08
cg02488349	7	ELMO1;ELMO1;ELMO1		0.000605	1.83E‐08
cg10232918	7	chr7:63634549‐63635889		0.032465	3.89E‐08
cg18209323	7	chr7:92672789‐92673016	Island	14.88551	7.62E‐08
cg09560488	8	OXR1;OXR1		0.034569	6.65E‐08
cg10206627	8	FAM66A	Body	0.005483	2.91E‐08
cg17327934	8	AF228730.1		0.001696	6.63E‐08
cg17811434	8	chr8:143645072‐143645673 (CpG island name)	Island	0.003196	2.00E‐09
cg25661986	8	TRPS1;TRPS1;TRPS1	Body	30.04272	9.12E‐08
cg10586836	10	ADD3‐AS1	Body	0.030346	3.96E‐08
cg06558491	11	AP003498.1		0.00836	1.39E‐08
cg10966661	11	11:134415126–134,416,887		0.001533	7.80E‐08
cg06789519	13	LINC00354;LINC00354		0.006288	8.71E‐08
cg11176151	14	chr14:104596580‐104598145		0.027727	3.65E‐08
cg15341132	15	RCCD1;RCCD1;RCCD1	Shore	0.018169	8.69E‐08
cg01232206	16	chr16:33038921‐33040607	Shelf	0.012142	2.64E‐08
cg21438754	17	AC087499.10		0.007483	4.38E‐08
cg26160150	18	chr18:15165109‐15165342 (CpG island name)	S_Shelf	0.004409	7.45E‐08
cg07280593	19	LILRA2;LILRA2;LILRA2;LILRA2	Exon	0.015024	4.67E‐08
cg22356268	19	ZNF274;ZNF274;ZNF274	Shore	0.019295	8.97E‐08
cg25422753	19	chr19:54775040‐54775040		0.014903	8.79E‐09
cg26341465	20	PHACTR3;PHACTR3;PHACTR3	Body	0.025945	1.60E‐08

### 3.4 Model discrimination and added model discrimination by methylation markers

In the case–control set, the methylation markers alone achieved similar AUC as the clinical risk score alone (0.69 vs. 0.68, respectively, Table 3). Adding the methylation score to the clinical risk score improved the model discrimination from 0.68 to 0.73 (*p* = 0.01). In the analyses stratified by Gleason score, similar findings were found for those diagnosed with Gleason score ≤6, 7, and ≥8 (Table 3). However, in the validation cohort, the methylation score alone achieved lower AUC compared to that from clinical risk score (0.73 vs. 0.80). There was also no improvement in AUC with the addition of the methylation score (AUC = 0.80 vs. 0.81). When stratified by Gleason score, there was a meaningful improvement (although not statistically significant) in AUC for those with Gleason score ≤6 (AUC = 0.91 [clinical risk score + methylation score] vs. 0.80 [clinical risk score alone], *p* = 0.60; Table 3).

**TABLE 3:** Odds ratios from conditional logistic regression and area under the ROC curve of the risk prediction models in the case–control dataset and in the validation cohort, overall and by Gleason score.

	Overall			Gleason ≤6			Gleason 7			Gleason ≥8		
	OR^a^	*p*	AUC	OR^a^	*p*	AUC	OR^a^	*p*	AUC	OR^a^	*p*	AUC
Case–control set												
Clinical risk score^b^	1.39 (1.29–1.50)	<0.01	0.68	1.27 (1.07–1.52)	<0.01	0.60	1.48 (1.21–1.80)	<0.01	0.65	1.42 (1.20–1.69)	<0.01	0.66
Methylation score^c^	1.16 (1.13–1.19)	<0.01	0.69	1.15 (1.08–1.22)	<0.01	0.66	1.12 (1.07–1.19)	<0.01	0.66	1.14 (1.09–1.19)	<0.01	0.68
Clinical risk score + methylation score	1.25 (1.15–1.35), 1.12 (1.09–1.16)	<0.01, <0.01	0.73*	1.19 (1.01–1.40), 1.14 (1.07–1.14)	<0.01, <0.01	0.67*	1.34 (1.09–1.64), 1.10 (1.04–1.16)	0.01, <0.01	0.71	1.28 (1.06–1.54), 1.11 (1.06–1.17)	0.01, <0.01	0.73
Validation cohort												
Clinical risk score^b^	3.61 (2.87–4.55)	<0.01	0.80	4.34 (2.79–6.75)	<0.01	0.80	2.96 (2.03–4.33)	<0.01	0.69	–	–	–
Methylation score^c^	1.28 (1.20–1.36)	<0.01	0.73	1.71 (1.51–1.93)	<0.01	0.87	1.04 (0.96–1.13)	0.37	0.53	–^d^	–	–
Clinical risk score + methylation score	3.24 (2.51–4.17), 1.13 (1.06–1.21)	<0.01, <0.01	0.81*	3.28 (1.92–5.61), 1.55 (1.36–1.76)	<0.01, <0.01	0.91	2.95 (2.01–4.32), 1.01 (0.93–1.09)	<0.01, 0.85	0.69	–	–	–

Abbreviations: AUC, area under the ROC curve; OR, odds ratio; *p*, *p* value.

^a^ OR is for one unit increase in the methylation score or the clinical score; it's magnitude should not be directly compared between clinical risk score and the methylation score, since the scores have different ranges. Rather, the *p* value and AUC are more informative for the purpose of our study.

^b^ Clinical risk score ranged from 4.2 to 12.0 in the entire case–control set and from 4.3 to 10.1 in the validation cohort. The ORs here represent one unit increase in the clinical risk score.

^c^ Methylation score ranged from −22.2 to −6.1 in the entire case–control set and −21.3 to −5.4 in the validation cohort. The ORs here represent one unit increase in the methylation score.

^d^ Individuals with Gleason ≥8 were not included in the validation cohort.

* *p* Value < 0.05 derived from a likelihood ratio test comparing the predictive model to the clinical risk score only model, which is equivalent to the comparison of AUCs from these two models.

### 3.5 Added model calibration by methylation markers

In the validation cohort, both clinical risk score alone and methylation marker score alone are well calibrated for the following groups of model‐predicted 10‐year metastasis risks: <5%, 5%–14.9%, and ≥15%. Observed cumulative incidence of metastasis was 1.6%, 9.6%, and 28.3% for these groups defined by clinical risk score alone, and 0%, 7.5%, and 32.8% for groups defined by methylation score alone.

**FIGURE 3:**
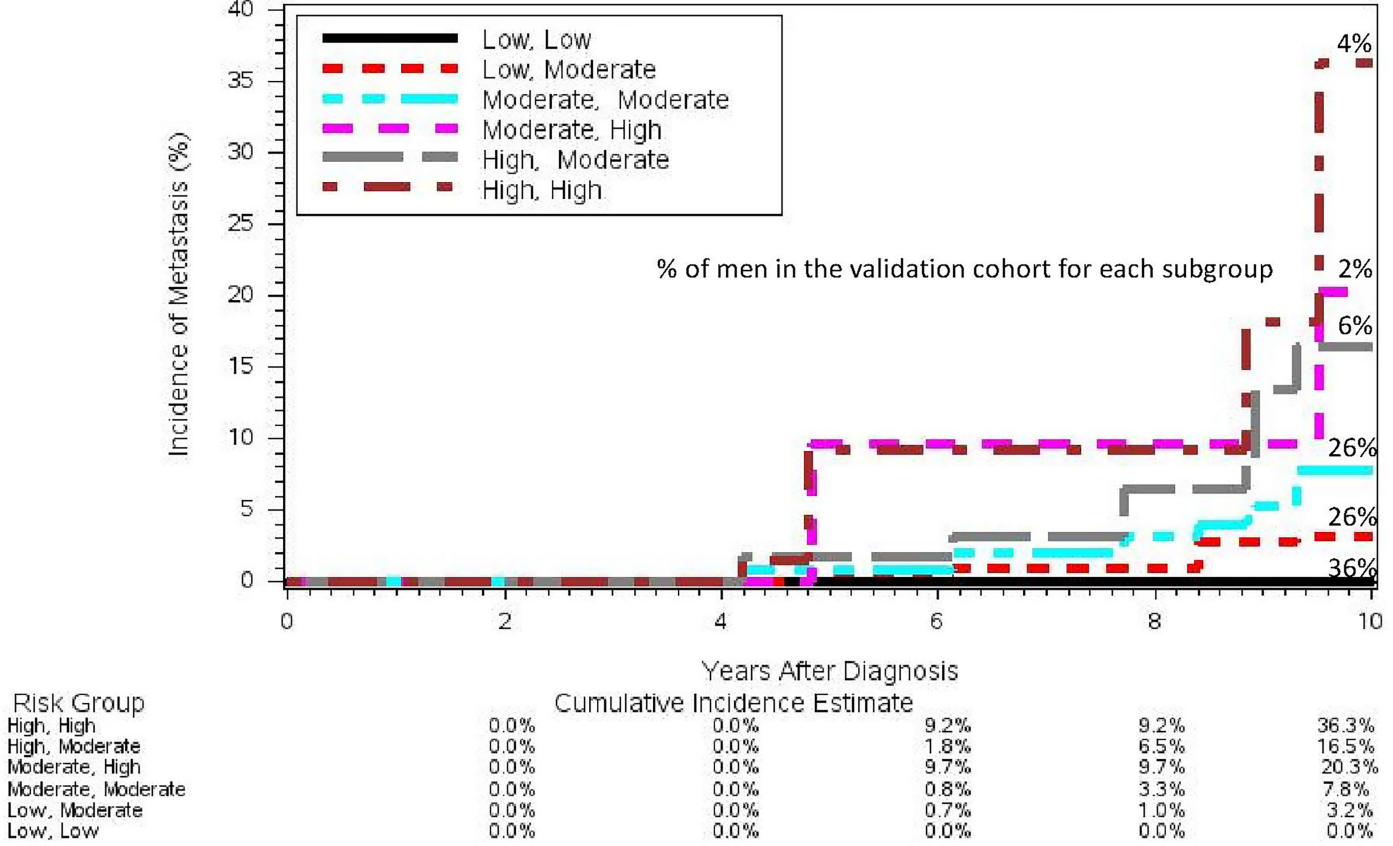
Ten‐year cumulative incidence for the model‐predicted risk categories determined by the predicted risk group from clinical risk score only and the predicted risk group from clinical risk score + methylation score. High: 15%+ 10‐year risk of metastasis; Moderate: 5‐14.9% 10‐year risk of metastasis; Low: <5% risk of metastasis. Predicted Risk Category was first labeled by the predicted risk group from the clinical risk score only (high, moderate, or low), followed by the predicted risk group when methylation score was added to the model (i.e., clinical risk score + methylation score). For example, the risk group [High, High] means that both the clinical risk score and the [clinical risk score+ methylation score] predicted high risk (15+%) for these individuals; while [High, Moderate] means that the clinical risk score only model predicted high risk (15+%) for these individuals, but the [clinical risk score+ methylation score] model predicted moderate risk (5‐14.9%) for these individuals.

When comparing model‐predicted risks from the model composed of clinical risk score only to that with the addition of the methylation score, both models assigned 65% of men into the same predicted risk groups, while 29% of men were moved to a higher risk category and 6% moved to a lower risk category. As shown in Figure 3, adding the methylation score only improved predicted calibration for 2% of the men (whose estimated risk increased from 5%–15% to >15% after adding methylation risk score [in Figure 3, moderate ≥ high] had an estimated 10‐year rate of 20.3% [high]) but worsen the predicted risk calibration for 33% of the men in the validation cohort (i.e., in Figure 3, low ≥ moderate [actual observed risk group: low] and high ≥ moderate [actual observed risk group: high]).

## 4 DISCUSSION

In this study, we identified 31 methylation markers after Bonferroni adjustment from genome‐wide methylation assay to be significantly associated with risk of metastasis among men with localized PCa that are untreated. Adding a risk score based on these 31 methylation markers to the clinical risk score in the risk prediction model moderately improved the predicted risk discrimination as suggested by the AUC in the case–control set (0.73 vs. 0.68), but not in the validation cohort (0.81 vs. 0.80). Further, adding the methylation score to clinical risk score did not enhance predicted risk calibration. Our findings do not support clinical use of these markers to improve overall risk prediction. However, our data suggest men with Gleason score ≤6 may be the exception. In this patient subset, methylation risk score alone achieved similar AUC (0.87) as the combined use of the two risk scores (0.91) and had a meaningful improvement in AUC in magnitude (from 0.80 to 0.91, although this change was not statistically significant). These findings suggest that the 31 methylation markers may help to identify patients with low grade Gleason score that are at higher risk of metastasis beyond clinical characteristics. Given the small event size of the validation cohort in this study, our findings will need to be confirmed in larger studies.

Although adding the methylation score to the clinical risk score did not improve predicted risk calibration in the validation cohort, it should be noted that the validation cohort only has 10 metastatic cases with successful methylation results. The potential of these methylation markers in improving predicted risk calibration should thus be evaluated in other larger cohorts of untreated men. However, it should be noted that when the addition of the methylation score changed the predicted risk category, they did further distinguish the metastasis risk within that predicted risk group. For example, for those predicted to have ≥15% 10‐year risk of metastasis by clinical risk score only, the addition of the methylation score further stratified this group into two groups (those that were downgraded vs. unchanged by the methylation score). Those who were downgraded did indeed had lower 10‐year cumulative incidence (16.5%) compared to those whose predicted risk group was not altered by methylation score (36.2%). This was also the case for those within the <5% predicted risk group by the clinical risk score. These findings support the value of these methylation markers in offering additional prognostic information beyond clinical risk factors, despite the calibration results in this study with the chosen predicted risk categories.

To our knowledge, this study is among the first to perform genome‐wide methylation profiling using diagnostic biopsy PCa tissue for the identification of prognostic methylation markers for risk of PCa metastasis in untreated men. As noted by a recent systematic review,^22^ existing literatures in the search of prognostic methylation markers have primarily focused on patients who received radical prostatectomy; a majority also used biochemical recurrence as the outcomes of interest rather than metastasis or lethal PCa. Many of these studies reported positive findings on prognostic methylation markers,^22^ including several recent studies with genome‐wide profiling that reported moderately improved the risk prediction^33^, ^34^, ^35^, ^36^, ^37^ for recurrent/lethal PCa. However, most of these studies did not evaluate predicted risk calibration.

The 31 methylation markers identified in this study may help inform the molecular mechanism of disease progression, and potentially inform the development of new therapeutic targets. Of the 31 methylation markers identified, six are within the gene body/coding regions. Some of these genes, namely TRPS1,^38^, ^39^ FAM66A,^40^ and PRDM16,^41^ have been implicated in prostate carcinogenesis, although research on these genes and PCa remain sparse. The roles of the other three genes (i.e., ADD3‐AS1, PHACTR3, and ALDH1L1‐AS2) in PCa development and progression have not been reported.

In addition, two markers are located in CpG islands, while six markers are located in the CpG shore and shelf regions. Sixteen of the 31 markers did not have a clear relation with CpG island or the gene body. This finding is similar to a prior study that reported that many of the differential methylated positions within cancer stem cells do not reside in CpG islands, but in shelf and open sea (sites outside of the CpG island regions^42^). Of note, methylation status was associated with a strong protective effect on metastasis for 28 out of the 31 markers. These findings may lead to novel hypotheses regarding the role of these genetic regions in PCa progression.

There are several limitations that should be considered when interpreting our results. First, there was a large attrition of study subjects for obtaining the methylation data. This was in part due to the retrospective nature of the study and in part due to the nature of small tumor size in the biopsy cores for those with very limited diseases. There is therefore potential selection bias that those with more “extensive” disease were more likely to be included in the final analytical datasets. Second, our algorithm may not have captured all metastases developed, and some men with metastasis may lack documentation in chart notes or were undiagnosed. Third, our cohorts of patients were diagnosed between 1997 and 2007, when the standard of care was a systematic sextant biopsy. Thus, it is possible that our cohort may be under‐staged due to low sampling. Fourth, the validation cohort was limited in sample size. The markers identified should be further examined in a larger validation cohort. Fifth, our methodology focused on CpG site‐level analyses. Thus, our findings do not rule out that possibility that a different approach (e.g., clustering supervised or unsupervised) may lead to the identification of methylation markers that may better improve the predicted risk discrimination and calibration.

Methylation markers provided limited increased ability to predict metastasis better than established clinical risk factors, with some potential benefit in patient risk stratification for those with Gleason score ≤6. These findings should be validated in larger cohorts. The methylation markers identified, however, may inform novel hypothesis in the roles of these genetic regions in metastasis development.

## AUTHOR CONTRIBUTIONS

**Chun R. Chao:** Conceptualization (lead); funding acquisition (lead); investigation (lead); methodology (lead); supervision (equal); writing – original draft (lead). **Jeff Slezak:** Data curation (equal); formal analysis (lead); investigation (equal); writing – review and editing (equal). **Kimberly Siegmund:** Methodology (equal); resources (supporting); writing – reivew and editing (supporting). **Kimberly Cannavale:** Project administration (lead); supervision (lead); validation (lead); writing – review and editing (equal). **Yu‐Hsiang Shu:** Formal analysis (supporting); methodology (supporting); writing – review and editing (equal). **Gary W. Chien:** Data curation (supporting); investigation (equal); validation (supporting); writing – review and editing (equal). **Xu‐Feng Chen:** Data curation (lead); project administration (supporting); writing – review and editing (equal). **Feng Shi:** Data curation (supporting); writing – review and editing (supporting). **Nan Song:** Data curation (supporting); writing – review and editing (supporting). **Stephen K. Van Den Eeden:** Data curation (lead); investigation (supporting); supervision (lead); writing – review and editing (equal). **Jiaoti Huang:** Conceptualization (supporting); data curation (lead); funding acquisition (supporting); supervision (lead); writing – review and editing (equal).

## FUNDING INFORMATION

This study was supported by National Cancer Institute grant R01CA181242 Identification of DNA methylation markers for risk of metastasis in localized prostate cancer.

## CONFLICT OF INTEREST STATEMENT

The authors declare the following financial interests/personal relationships which may be considered as potential competing interests: Chun R. Chao received research funding from Merck & Co. for research projects unrelated to this work. Jeff Slezak received research funding from Pfizer, Dynavax Technologies Corp. and ALK‐Abello for research projects unrelated to this work. Jiaoti Huang is a consultant for or owns shares in the following companies: Amgen, Artera, Kingmed Diagnostics, MoreHealth, OptraScan, York Biotechnology, Genecode, Seagen Inc. and Sisu Pharma, and received grants from Zenith Epigenetics, BioXcel Therapeutics, Inc., Dracen Pharmaceuticals, and Fortis Therapeutics.

## ETHICAL APPROVAL

KPSC's and KPNC's Institutional Review Boards approved this study and waived the requirement for informed consent.

## Supporting information


Figure S1.

## Data Availability

Anonymized data that support the findings of this study may be made available from the corresponding author on reasonable request from qualified researchers with documented evidence of training for human subjects protections.
