# Supplementary material for: Genome‐wide methylation profiling of diagnostic tumor specimens identified DNA methylation markers associated with metastasis among men with untreated localized prostate cancer
Source: Cancer Med. 2023 Sep 11;12(18):18837–49. doi: 10.1002/cam4.6507 (PMC10557825; doi:10.1002/cam4.6507)
Supplement: Supplementary file 1 — Figure S1. [file CAM4-12-18837-s001.pdf]

Supplemental Figure

Bi-directional genome-wide Manhattan plot displaying differentially-methylated CpG sites associated with metastasis

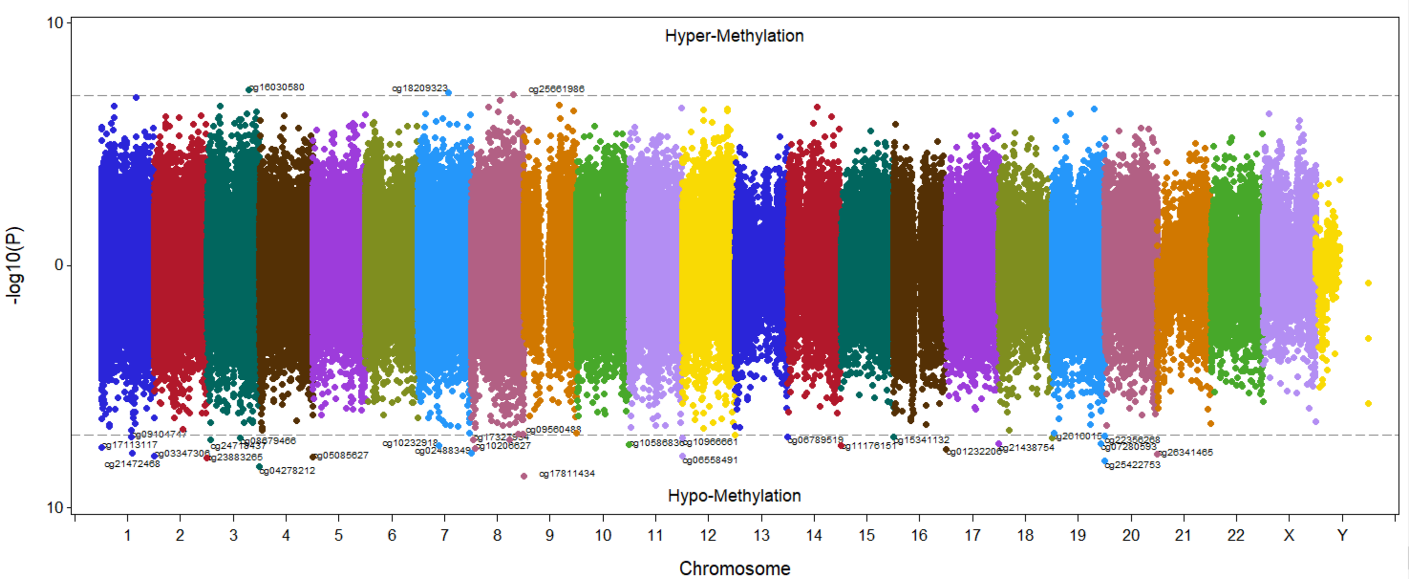

Dotted line represents p-value of -log10(7)
